# Supplementary material for: The human HELLS chromatin remodelling protein promotes end resection to facilitate homologous recombination and contributes to DSB repair within heterochromatin
Source: Nucleic Acids Res. 2019 Dec 5;48(4):1872–85. doi: 10.1093/nar/gkz1146 (PMC7038987; doi:10.1093/nar/gkz1146)
Supplement: gkz1146_Supplemental_Files [file gkz1146_supplemental_files.zip › Revised_supplementary_figures_.pdf]

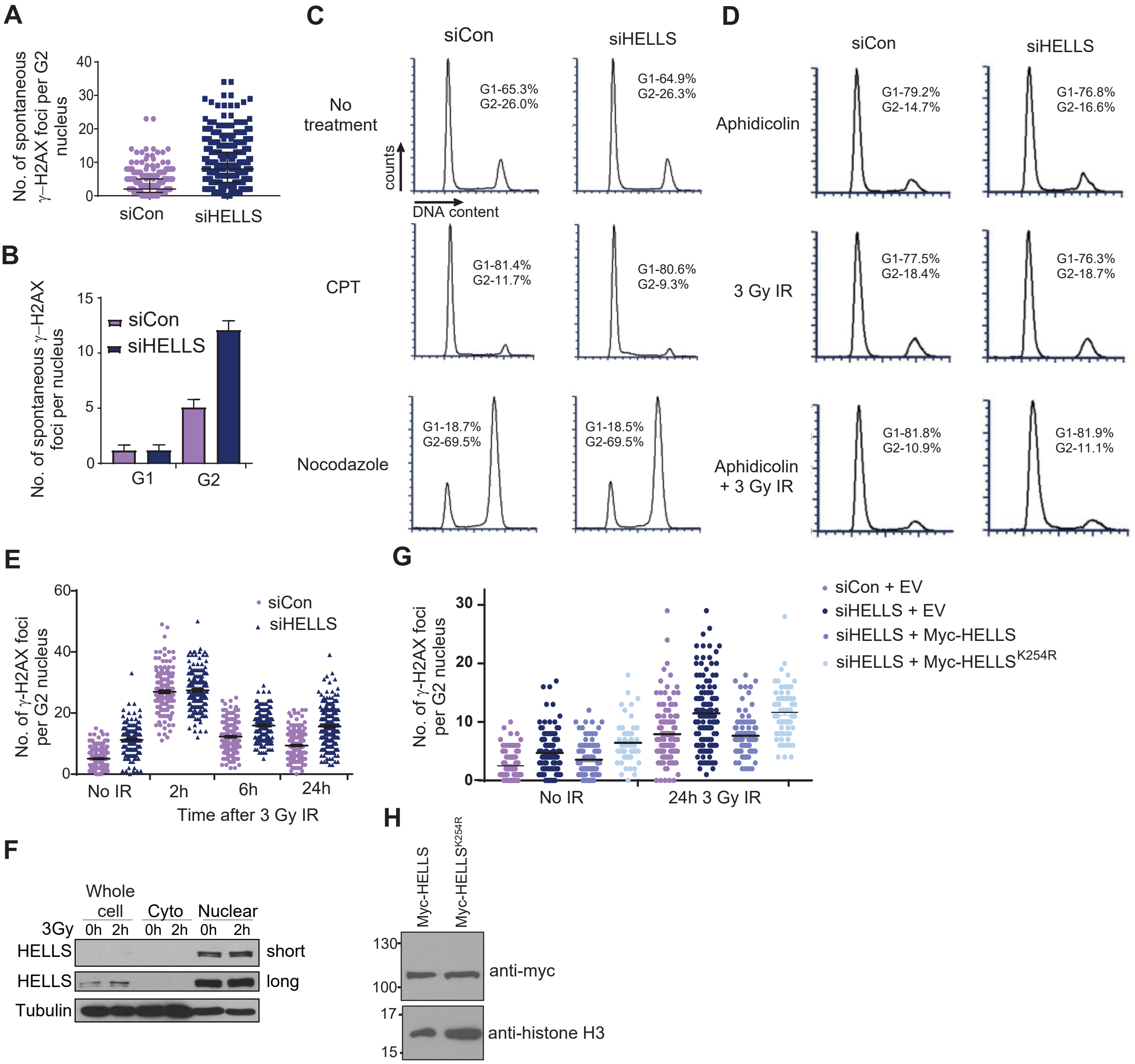

Kollarovic_Fig S1

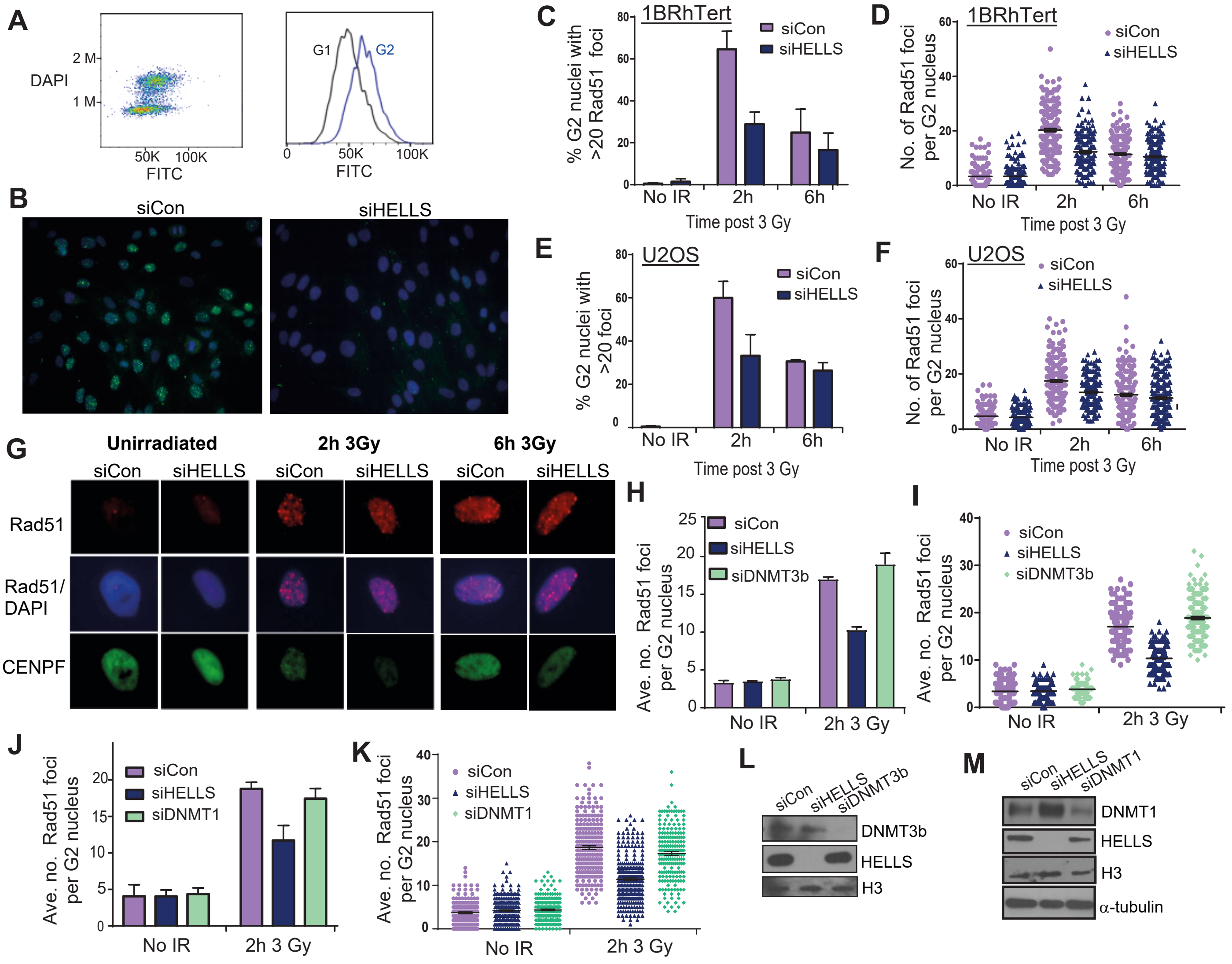

Kollarovic_FigS2

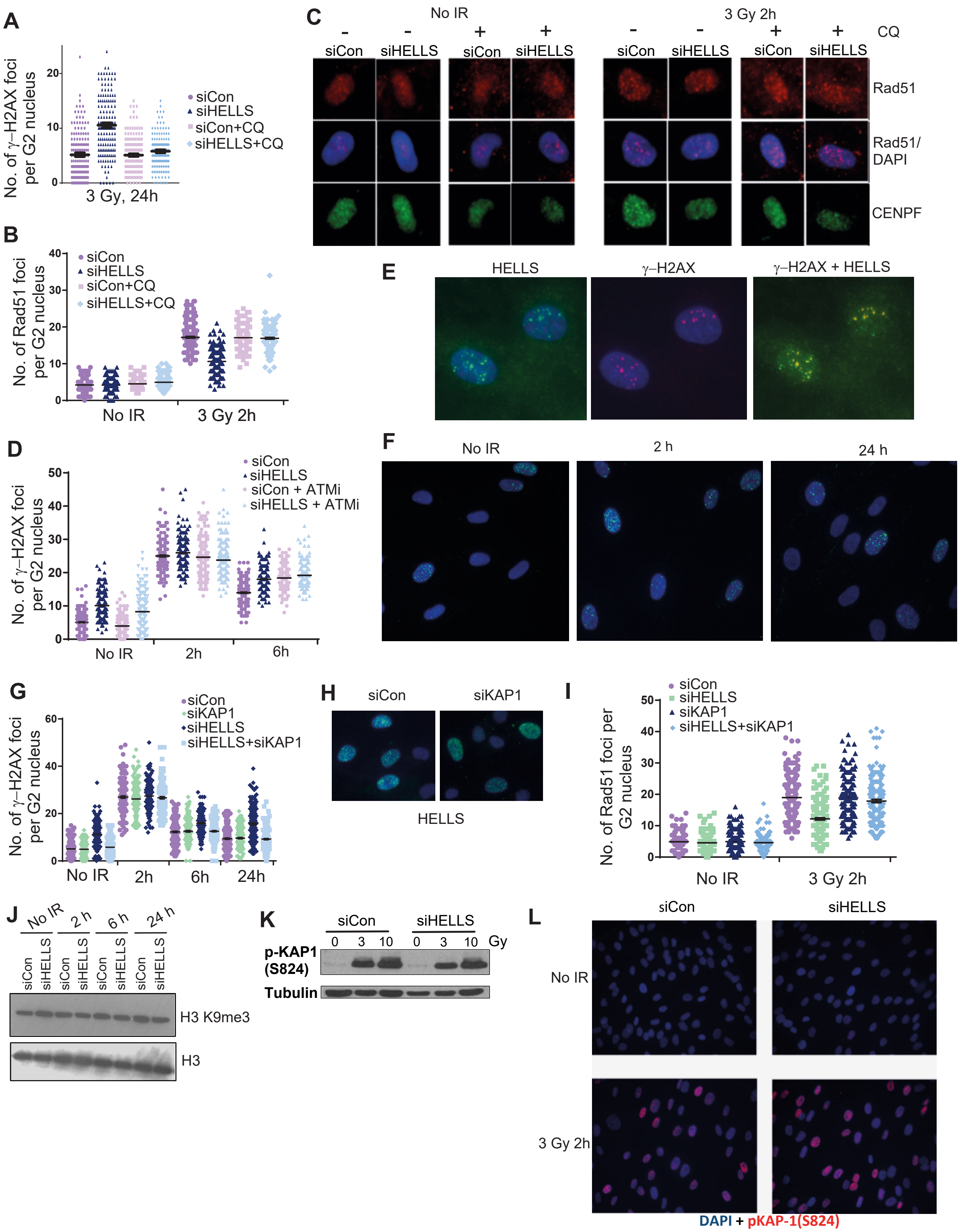

Kollarovic_FigS3

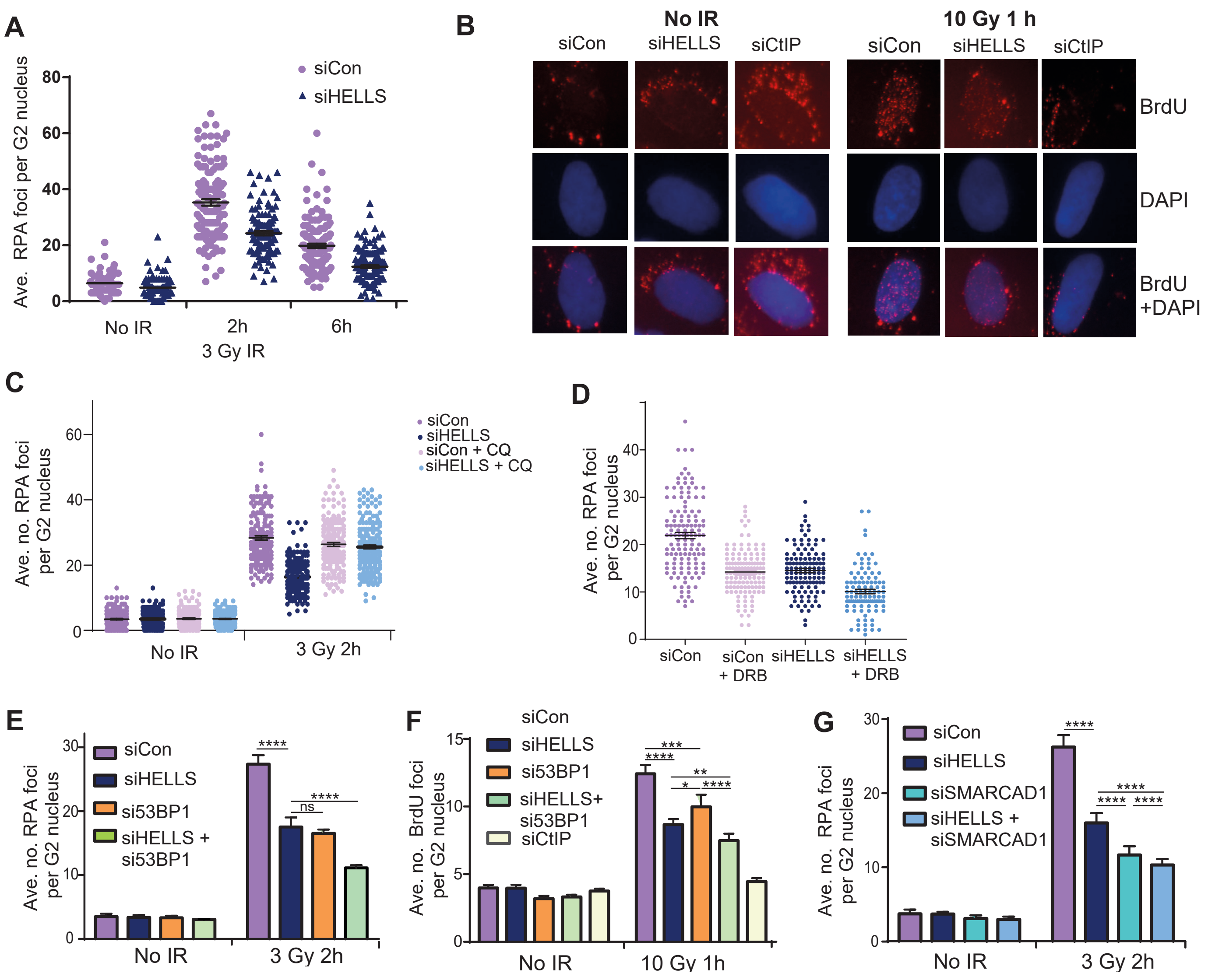

Kollarovic_FigS4

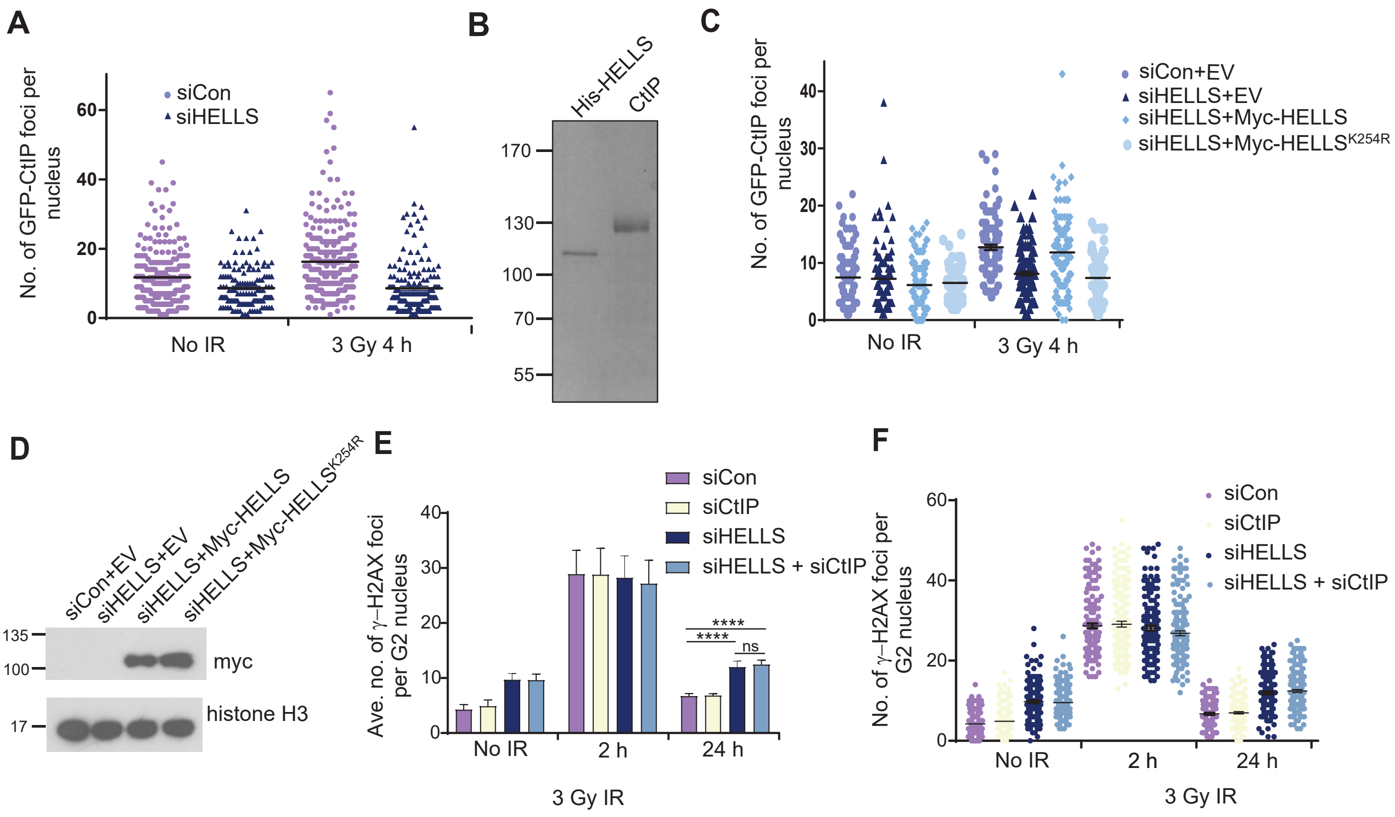

Kollarovic_Fig S5

Supplementary Figure Legends

Figure S1. HELLS promotes genome stability in undamaged cells and following IR

(A) Counts of spontaneous γ-H2AX foci per G2 nucleus in 1BR-hTert cells transfected with siCon or siHELLS. Data from six independent experiments with median and interquartile range shown. **(B)** Ave. number of spontaneous γ-H2AX foci in G0/G1 serum-starved (5 independent experiments) or G2 (data as Fig1C) nuclei in 1BR-hTert cells transfected with siCon or siHELLS. **(C)** Cell cycle and checkpoint analysis by FACS of 1BR-hTert cells transfected with indicated siRNA. 0.5 µM CPT or 100 ng/ml nocodozole were added at time of siRNA transfection and samples collected after 24 hours. **(D)** Cell cycle analysis by FACS of 1BR-hTert cells transfected with the indicated siRNA 24 h prior to irradiation. 6 µM aphidicolin was added at the time of irradiation and samples collected 24 h after irradiation. **(E)** γ-H2AX foci counts per G2 nucleus in 1BR-hTert cells transfected with siCon or siHELLS at indicated time points following 3 Gy IR. Data is combined from three independent experiments and mean +/- sem shown. **(F)** Western blot analysis of distribution of HELLS in cytoplasmic and nuclear fractions prepared from undamaged 1BR-hTert cells, or 2 h following 3 Gy IR. Alpha tubulin is used as a loading control. **(G)** Counts of the number of IR-induced γ-H2AX foci per nucleus in U2OS cells transfected with siCon or HELLS siRNA and constructs expressing siRNA-resistant wt or ATPase mutant HELLS. Combined data from at least three independent experiments and mean and sem shown. **(H)** Western blot analysis of myc-HELLS wt and Myc-$HELLS^{K254R}$ mutant proteins in extracts prepared from U2OS cells transfected with indicated siRNA and expression plasmid.

Figure S2. HELLS depletion results in defects in the homologous recombination pathway of DSBR

(A) FACS analysis of HELLS expression in G1 vs G2 using anti-HELLS (FITC) and DAPI staining. **(B)** Anti-HELLS and DAPI immunofluorescent images of 1BR-hTert cells transfected with siCon or siHELLS. **(C)** % of G2 nuclei with greater than 20 Rad51 foci in 1BR-hTert cells transfected with siCon or siHELLS. (**D)** Number of Rad51 foci per G2 nucleus in 1BR-hTert cells transfected with siCon or siHELLS. **(E)** % of G2 nuclei with greater than 20 Rad51 foci in U2OS cells transfected with indicated siRNA. **(F)** Number of Rad51 foci per G2 nucleus in U2OS cells transfected with siCon or siHELLS. **(G)** Representative immunofluorescent images of Rad51 foci in U2OS cells transfected with the indicated siRNA following 3Gy IR. **(H)** Quantification of IR-induced Rad51 foci in G2 nuclei of 1BR-hTert cells transfected with indicated siRNA. **(I)** Number of Rad51 foci per G2 nucleus in 1BR-hTert cells transfected indicated siRNA. **(J)** Quantification of IR-induced Rad51 foci in G2 nuclei of 1BR-hTert cells transfected with indicated siRNA. **(K)** Number of Rad51 foci per G2 nucleus in 1BR-hTert cells transfected indicated siRNA. **(L)** Western blot analysis of efficiency of DNMT3b and HELLS depletion by siRNA from nuclear extracts prepared from 1BR-hTert cells. Histone H3 used as a loading control. **(M)** Western blot analysis of efficiency of DNMT1 and HELLS depletion by siRNA from nuclear extracts prepared from 1BR-hTert cells. Alpha tubulin used as a loading control. For panels C,D, E, F, H, I J and K data shown are mean and sem from three independent experiments.

Figure S3. HELLS facilitates homologous recombination within heterochromatin

(A) Individual counts of number of γ-H2AX foci per G2 nucleus in 1BR-hTert cells transfected with siCon or siHELLS in the presence or absence of chloroquine 24 h after 3 Gy IR. **(B)** Individual counts of number of spontaneous Rad51 foci per G2 nucleus in 1BR-hTert cells transfected with siCon or siHELLS in the presence or absence of chloroquine 2 h after 3 Gy IR. **(C)** Representative immunofluorescent images of Rad51 foci in 1BR-hTert cells transfected the indicated siRNA with or without addition of 40 µg/ml chloroquine 2 h prior to irradiation. **(D)** Individual counts of number of γ-H2AX foci per G2 nucleus in 1BR-hTert cells transfected with siCon or siHELLS. Where indicated, 10 µM ATMi was added 1 h prior to irradiation. **(E)** Additional representative immunofluorescent images of HELLS and γ-H2AX

foci colocalisation, associated with Figs 3E and 3F. **(F)** Immunofluorescence images of endogenous HELLS indicating that its expression and localisation is unchanged after 3 Gy IR. **(G)** Rescue of clearance of γ-H2AX foci by KAP-1 depletion following 3 Gy irradiation in 1BR-hTert cells. Data for siCon and siHELLS is the same as that shown in Figure 1F and S1E. **(H)** Representative immunofluorescent images of 1BR-hTert cells indicating that the punctate nuclear localisation of HELLS is not affected by KAP-1 depletion. **(I)** Number of Rad51 foci formation per nucleus following 3 Gy IR in 1BR-hTert cells transfected with the indicated siRNA. **(J)** Western blot analysis of H3 K9me3 in 1BR-hTert cells transfected with siCon or siHELLS, at indicated time points following 3 Gy IR. Total H3 is used as a loading control. **(K)** Western blot analysis of phosphorylated (S824) KAP-1 in 1BR-hTert cells transfected with siCon or siHELLS 30 min following 3 Gy or 10 Gy IR. **(L)** Immunofluorescent images of pKAP- 1 (S824) in 1BR-hTert cells transfected with siCon or siHELLS, before or 2 h following 3 Gy IR. In panels A, B,D, G and I data are combined from three independent experiments and mean and sem indicated.

Figure S4. HELLS promotes end resection
(A) Individual counts of number of RPA foci per G2 nucleus in 1BR-hTert cells transfected with siCon or siHELLS. **(B)** Representative immunofluorescent images of BrdU foci 1 h after 10 Gy IR under non-denaturing conditions in 1BR-hTert cells transfected with the indicated siRNA. **(C)** Individual counts of number of RPA foci per G2 nucleus in 1BR-hTert cells transfected with siCon or siHELLS in the presence or absence of chloroquine 2 h after 3 Gy IR. **(D)** Individual counts of number of RPA foci per G2 nucleus in 1BR-hTert cells transfected with siCon or siHELLS in the presence or absence of DRB 2 h after 3 Gy IR. **(E)** Quantification of RPA foci per G2 nucleus 2 h after 3 Gy, in 1BR-hTert cells transfected with Control, HELLS and 53BP1 siRNAs. **(F)** Quantification of BrdU foci per nucleus 1 h after 10 Gy, detected under non-denaturing conditions, in 1BR-hTert cells transfected with Control, HELLS, CtIP and 53BP1 siRNAs. **(G)** Quantification of RPA foci per G2 nucleus 2 h after 3 Gy, in 1BR-hTert cells transfected with Control, HELLS and SMARCAD1 siRNAs. **** indicates a p-value of <0.0001, *** indicates p-value of <0.001, ** indicates p-value of <0.01, ** indicates p-value of <0.1 and ns indicates not significant by unpaired two-tailed t-test analysis. For panels A, C, D, E, F and G, data shown are the mean and sem from three independent experiments.

Figure S5. HELLS interacts with CtIP and contributes to its accumulation at IR-induced breaks
(A) Individual counts of number of CtIP-GFP per nucleus 4 h following 3 Gy IR in U2OS cells transfected with CtIP-GFP expressing plasmid and the indicated siRNA. **(B)** Coomassie stained SDS-PAGE gel showing 500 ng of purified recombinant His-HELLS and CtIP proteins. **(C)** Individual counts of the number of IR-induced CtIP-GFP foci per nucleus in U2OS cells transfected with CtIP-GFP expressing plasmid, HELLS siRNA and constructs expressing siRNA-resistant wt or ATPase mutant HELLS. **(D)** Western blot analysis of myc-HELLS wt and myc-HELLS K254R proteins in chromatin fraction of U2OS cells transfected with the indicated siRNA and plasmid. **(E)** Quantification of clearance of γ-H2AX foci in G2 nuclei following 3 Gy IR in 1BR-hTert cells transfected with Control, CtIP and HELLS siRNAs. **(F)** Individual counts of number of γ-H2AX foci per G2 nucleus following 3 Gy IR in 1BR-hTert cells transfected with Control, CtIP and HELLS siRNAs. **** indicates a p-value of <0.0001, and ns indicates not significant by unpaired two-tailed t-test analysis. In panels A, C, E and F data were combined from three independent experiments with the mean and sem shown.
