# Supplementary material for: The human HELLS chromatin remodelling protein promotes end resection to facilitate homologous recombination and contributes to DSB repair within heterochromatin
Source: Nucleic Acids Res. 2019 Dec 5;48(4):1872–85. doi: 10.1093/nar/gkz1146 (PMC7038987; doi:10.1093/nar/gkz1146)
Supplement: gkz1146_Supplemental_Files [file gkz1146_supplemental_files.zip › Revised Supplementary Figures gkz1146.zip!/5137_c9cb052bef23499ebcff067d6e4974e3f533162854f3745589f22786b87412ca_files/index.html]

index


# 5137 c9cb052bef23499ebcff067d6e4974e3f533162854f3745589f22786b87412ca

Unknown

**A**

**C**

**D**

siCon

siHELLS

siCon

s

2

siHELLS

u

40

o

r G

G1-64.9%

e

e

G1-65.3%

n

30

G2-26.3%

G1-79.2%

G1-76.8%

ta

i p

s

No

G2-26.0%

G2-14.7%

G2-16.6%

n

c

u

o

le

treatment

20

Aphidicolin

p

fo

c

X

un

ts

f s

A

10

n

2

u

. o

o

o

H

c

N

g-

0

siCon

DNA content

siHELLS

**B**

G1-77.5%

G1-76.3%

G1-81.4%

G1-80.6%

G2-18.4%

G2-18.7%

X

CPT

G2-11.7%

G2-9.3%

3 Gy IR

A

15

2H

siCon

g-

su

s

10

siHELLS

u

le

o

c

e

u

n

r n

ta

e

5

no i p

G1-18.7%

G1-18.5%

p

c

G1-81.8%

G1-81.9%

G2-69.5%

G2-69.5%

fo

G2-10.9%

G2-11.1%

f s

0

Nocodazole

Aphidicolin

. oo

G1

G2

+ 3 Gy IR

N

**E**

**G**

60

siCon + EV

siCon

i

**30**

i

c

siHELLS + EV

c

s

s

siHELLS

fo

u

siHELLS + Myc-HELLS

fo

u

X

le

X

le 40

A

c

A

c

2

u **20**

siHELLS + Myc-HELLSK254R

2

u

n

n

g-H

2

g-H

2

f

f

20

r G

r G

. o

e

. o

e

o

**10**

p

o

p

N

N

0

**0**

No IR

2h

6h

24h

No IR

24h 3 Gy IR

Time after 3 Gy IR

**H**

R452

**F**

K

S

S

L

L

L

L

E

E

Whole

-H

-H

c

c

cell

Cyto Nuclear

y

y

3Gy

M

0h 2h

0h 2h 0h

2h

M

HELLS

short

130

anti-myc

HELLS

long

100

Tubulin

17

anti-histone H3

15

Kollarovic\_Fig S1

**80** 1BRhTert

**C**

**D**

**A**

1BRhTert

i

**60**

i

siCon

ith

c

c

s

siCon

G1

G2

siHELLS

u

2 M

siHELLS

**60**

i w

fo

fo

le

1

1

c

DAPI

le

5

**40**

c

5

u

d

da

1 M

u

**40**

n

n

a

2

2

R

f R

**20**

G

0

r G

**20**

. o

e

0

50K

50K

100K

o

100K

%

>2

p

N

FITC

FITC

**0**

**0**

No IR

2h

6h

**B**

No IR

2h

6h

siCon

siHELLS

Time post 3 Gy

Time post 3 Gy

**E**

**80**

U2OS

**F**

U2OS

**60**

siCon

siCon

i

ith

c

siHELLS

**60**

siHELLS

s

i w

i

fo

u

le

c

1

le **40**

c

5

c

fo **40**

u

d

u

0

a

n

n

2

2

>2

**20**

f R

**20**

G

. o

r G

%

o

ep

**0**

N

**0**

**Unirradiated**

**2h 3Gy**

**6h 3Gy**

No IR

2h

6h

No IR

2h

6h

**G**

siCon

siHELLS

siCon

siHELLS

siCon

siHELLS

Time post 3 Gy

Time post 3 Gy

**H**

**I**

25

**40**

Rad51

siCon

i

i

c

siCon

c

20

siHELLS

fo

s

siHELLS

**30**

fo

su

siDNMT3b

1

u

1

5

le

siDNMT3b

Rad51/

5

le 15

d

c

d

c

a

u

DAPI

a

u

**20**

n

n 10

. R

2

. R

2

o

o

r G **10**

CENPF

r G 5

. n

e

. n

e

e

e

p

v

p

v

A

A

0

**0**

No IR

2h 3 Gy

No IR

2h 3 Gy

**J**

i

**K** i

c

**40**

c

siCon

**M**

s 20

fo

fo

s

siCon

1

u

siCon

u

siDNMT1

siHELLS

**L**

siHELLS

1

siHELLS

5

le

**30**

5

le

d

c

siDNMT3b

siHELLS

siCon

15

siDNMT1

d

c

siDNMT1

DNMT1

a

u

a

u

DNMT3b

n

n **20**

HELLS

2 10

. R

. R

2

o

o

HELLS

r G

H3

r G

. n

e

5

. n

e **10**

e

H3

e

p

v

p

v

a-tubulin

0

A

A

**0**

No IR

2h 3 Gy

No IR

2h 3 Gy

Kollarovic\_FigS2

No -

**A**

IR

3 Gy 2h

**C** -

-

+

+

-

-

+

+

CQ

siCon

siHELLS

siCon

siHELLS

siCon

siHELLS

siCon

siHELLS

ic s **20**

fo

u

Rad51

X

le

siCon

A

c

siHELLS

2

u

H

n **10**

siCon+CQ

g-

2

siHELLS+CQ

Rad51/

f

r G

DAPI

. o

e

o

p

**0**

N

3 Gy, 24h

CENPF

**B**

**40**

ic

siCon

s

siHELLS

**E**

HELLS

g-H2AX

g-H2AX + HELLS

fo

u **30**

1

le

siCon+CQ

5

c

d

u

siHELLS+CQ

a

n **20**

2

f R

. o

r G **10**

o

ep

N

**0**

No IR

3 G

y 2

h

**F**

No IR

2 h

**D**

24 h

siCon

**50**

siHELLS

ic

siCon + ATMi

s **40**

fo

u

siHELLS + ATMi

X

le

A

c **30**

2

u

H

n

g-

2 **20**

f

r G

. o

ep **10**

oN

**0**

No IR

2h

6h

**G**

**H**

siCon

siCon

siKAP1

**I**

siCon

**50**

siHELLS

i

siKAP1

r

siKAP1

**60**

c

e

siHELLS

s

**40**

siHELLS+siKAP1

fo

u

i p

siHELLS+siKAP1

c

X

le

s

A

c

u

**40**

fo

**30**

2

u

1

le

H

c

n

5d u **20**

g-

2

a

n

f

**20**

2

r G

HELLS

f R

. o

e

G **10**

o

p

. o

N

o

**0**

N

**0**

No IR

2h

6h

24h

No IR

3 Gy 2h

**J**

siCon

siHELLS

**L**

No IR

6 h

24 h

2 h

**K**

siCon

siHELLS

S

S

S

S

0

3

10

0

3

10 Gy

L

L

L

L

**p-KAP1**

n

L

n

L

n

L

n

L

o

E

o

E

o

E

o

E

**(S824)**

iC

iH

iC

iH

iC

iH

iC

iH

No IR

s

s

s

s

s

s

s

s

**Tubulin**

H3 K9me3

H3

3 Gy 2h

**DAPI + pKAP-1(S824)**

Kollarovic\_FigS3

**A**

**B**

**No IR**

**10 Gy 1 h**

siCon

siHELLS

siCtIP

siCon

siHELLS

siCtIP

su

siCon

**80**

lecu

siHELLS

BrdU

n2 **60**

r Ge

i p **40**

c

DAPI

foAP **20**

. Re

BrdU

vA **0**

+DAPI

No IR

2h

6h

3 Gy IR

**C**

**D**

siCon

60

siHELLS

ic

i

s

siC

i on +

C

+ Q

40

c

u

siHELLS + CQ

o

s

fo

f

le

u

A

e

c

A

l

P

c

u

30

40

P

u

n

R

. R

2

n

.

o

o

2

r G

n

G

. n

20

e

.

r

ev

20

e

e

p

v

p

A

A

10

0

0

No IR

3 Gy 2h

siCon

siCon siHELLS siHELLS

+ DRB

+ DRB

siCon

30

**E**

**F**

**G**

siCon

\*\*\*\*

30

15

siCon

\*\*\*\*

siHELLS

\*\*\*

i

i

\*\*\*\*

i

siHELLS

c

s

c

s

si53BP1

c

siHELLS

\*\*

s

fo

u

fo

u

\* \*\*\*\*

fo

u 20

siSMARCAD1

\*\*\*\*

A

le

\*\*\*\*

ns

U

le10

siHELLS+

A

le

c 20

si53BP1

\*\*\*\* \*\*\*\*

P

c

u

rd

u

si53BP1

P

c

siHELLS +

u

. R

n

siHELLS +

n

siCtIP

n

siSMARCAD1

2

. B

2

. R

o

si53BP1

o

o

2 10

. n

r G 10

r G 5

. n

. n

r G

e

e

e

e

e

e

v

p

v

p

v

p

A

A

A

0

0

0

No IR

3 Gy 2h

No IR

10 Gy 1h

No IR

3 Gy 2h

Kollarovic\_FigS4

**A**

**B**

**C**

re

r

LLS

E

i p

siCon+EV

e

c

siCon

tIP

**40**

siHELLS+EV

i p

is-H

**60**

C

fo

c

siHELLS

H

s

siHELLS+Myc-HELLS

fo

tIP

u

s

170

siHELLS+Myc-HELLSK254R

-C

le **30**

tIP

u

c

P

u

le **40**

-C

F

n

c

P

u

F

n

f G

**20**

130

. o

f G

**20**

o

. o

N

**10**

o

100

N

**0**

70

**0**

No IR

3 Gy 4 h

No IR

3 Gy 4 h

55

**D**

**E**

**F**

254R

K

siCon

LLS

LLS

60

siCon

E

E

i

40

siCtIP

c

re

siCtIP

V

yc-H yc-H

siHELLS

fo

i p

V

+E

+M

+M

X

s

siHELLS

c

u 30

siHELLS + siCtIP

A

s

LLS LLS LLS

2

le

fo

u 40

siHELLS + siCtIP

on+EE

E

E

H

c

X

u

le

A

c

siC siH siH

siH

g-

20

2

u

135

f

n

\*\*\*\*

2

\*\*\*\*

H

n

ns

myc

. o

g-

2

100

o

r G

20

G

e 10

f

. n

p

. o

e

o

histone H3

v

17

A

N

0

No IR

0

2 h

24 h

No IR

2 h

24 h

3 Gy IR

3 Gy IR

Kollarovic\_Fig S5

**Supplementary Figure Legends**

**Figure S1. HELLS promotes genome stability in undamaged cells and following IR**

**(A)** Counts of spontaneous γ-H2AX foci per G2 nucleus in 1BR-hTert cells transfected with siCon or siHELLS. Data from six independent experiments with median and interquartile range shown. **(B)** Ave. number of spontaneous γ-H2AX foci in G0/G1 serum-starved (5 independent experiments) or G2 (data as Fig1C) nuclei in 1BR-hTert cells transfected with siCon or siHELLS. **(C)** Cell cycle and checkpoint analysis by FACS of 1BR-hTert cells transfected with indicated siRNA. 0.5 μM CPT or 100 ng/ml nocodozole were added at time of siRNA transfection and samples collected after 24 hours. **(D)** Cell cycle analysis by FACS of 1BRhTert cells transfected with the indicated siRNA 24 h prior to irradiation. 6 μM aphidicolin was added at the time of irradiation and samples collected 24 h after irradiation. **(E)** γ-H2AX foci counts per G2 nucleus in 1BR-hTert cells transfected with siCon or siHELLS at indicated time points following 3 Gy IR. Data is combined from three independent experiments and mean +/-

sem shown. **(F)** Western blot analysis of distribution of HELLS in cytoplasmic and nuclear fractions prepared from undamaged 1BR-hTert cells, or 2 h following 3 Gy IR. Alpha tubulin is used as a loading control. **(G)** Counts of the number of IR-induced γ-H2AX foci per nucleus in U2OS cells transfected with siCon or HELLS siRNA and constructs expressing siRNA-resistant wt or ATPase mutant HELLS. Combined data from at least three independent experiments and mean and sem shown. **(H)** Western blot analysis of myc-HELLS wt and Myc-HELLSK254R mutant proteins in extracts prepared from U2OS cells transfected with indicated siRNA and expression plasmid.

**Figure S2. HELLS depletion results in defects in the homologous recombination** **pathway of DSBR**

**(A)** FACS analysis of HELLS expression in G1 vs G2 using anti-HELLS (FITC) and DAPI staining. **(B)** Anti-HELLS and DAPI immunofluorescent images of 1BR-hTert cells transfected with siCon or siHELLS. **(C)** % of G2 nuclei with greater than 20 Rad51 foci in 1BR-hTert cells transfected with siCon or siHELLS. (**D)** Number of Rad51 foci per G2 nucleus in 1BR-hTert cells transfected with siCon or siHELLS. **(E)** % of G2 nuclei with greater than 20 Rad51 foci in U2OS cells transfected with indicated siRNA. **(F)** Number of Rad51 foci per G2 nucleus in U2OS cells transfected with siCon or siHELLS. **(G)** Representative immunofluorescent images of Rad51 foci in U2OS cells transfected with the indicated siRNA following 3Gy IR. **(H)** Quantification of IR-induced Rad51 foci in G2 nuclei of 1BR-hTert cells transfected with indicated siRNA.  **(I)** Number of Rad51 foci per G2 nucleus in 1BR-hTert cells transfected indicated siRNA. **(J)** Quantification of IR-induced Rad51 foci in G2 nuclei of 1BR-hTert cells transfected with indicated siRNA.  **(K)** Number of Rad51 foci per G2 nucleus in 1BR-hTert cells transfected indicated siRNA.  **(L)** Western blot analysis of efficiency of DNMT3b and HELLS

depletion by siRNA from nuclear extracts prepared from 1BR-hTert cells. Histone H3 used as a loading control. **(M)** Western blot analysis of efficiency of DNMT1 and HELLS depletion by siRNA from nuclear extracts prepared from 1BR-hTert cells. Alpha tubulin used as a loading control. For panels C,D, E, F, H, I J and K data shown are mean and sem from three independent experiments.

**Figure S3. HELLS facilitates homologous recombination within heterochromatin** **(A)** Individual counts of number of γ-H2AX foci per G2 nucleus in 1BR-hTert cells transfected with siCon or siHELLS in the presence or absence of chloroquine 24 h after 3 Gy IR. **(B)** Individual counts of number of spontaneous Rad51 foci per G2 nucleus in 1BR-hTert cells transfected with siCon or siHELLS in the presence or absence of chloroquine 2 h after 3 Gy IR. **(C)** Representative immunofluorescent images of Rad51 foci in 1BR-hTert cells transfected the indicated siRNA with or without addition of 40 μg/ml chloroquine 2 h prior to irradiation. **(D)** Individual counts of number of γ-H2AX foci per G2 nucleus in 1BR-hTert cells transfected with siCon or siHELLS. Where indicated, 10 μM ATMi was added 1 h prior to irradiation. **(E)** Additional representative immunofluorescent images of HELLS and γ-H2AX

foci colocalisation, associated with Figs 3E and 3F. **(F)** Immunofluorescence images of endogenous HELLS indicating that its expression and localisation is unchanged after 3 Gy IR.

**(G)** Rescue of clearance of γ-H2AX foci by KAP-1 depletion following 3 Gy irradiation in 1BRhTert cells. Data for siCon and siHELLS is the same as that shown in Figure 1F and S1E. **(H)** Representative immunofluorescent images of 1BR-hTert cells indicating that the punctate nuclear localisation of HELLS is not affected by KAP-1 depletion. **(I)** Number of Rad51 foci formation per nucleus following 3 Gy IR in 1BR-hTert cells transfected with the indicated siRNA. **(J)** Western blot analysis of H3 K9me3 in 1BR-hTert cells transfected with siCon or siHELLS, at indicated time points following 3 Gy IR. Total H3 is used as a loading control. **(I)** Western blot analysis of phosphorylated (S824) KAP-1 in 1BR-hTert cells transfected with siCon or siHELLS 30 min following 3 Gy or 10 Gy IR. **(L)** Immunofluorescent images of pKAP-1 (S824) in 1BR-hTert cells transfected with siCon or siHELLS, before or 2 h following 3 Gy IR. In panels A, B,D, G and I data are combined from three independent experiments and mean and sem indicated.

**Figure S4. HELLS promotes end resection** **(A)** Individual counts of number of RPA foci per G2 nucleus in 1BR-hTert cells transfected with siCon or siHELLS. **(B)** Representative immunofluorescent images of BrdU foci 1 h after 10 Gy IR under non-denaturing conditions in 1BR-hTert cells transfected with the indicated siRNA.

**(C)** Individual counts of number of RPA foci per G2 nucleus in 1BR-hTert cells transfected with siCon or siHELLS in the presence or absence of chloroquine 2 h after 3 Gy IR. **(D)** Individual counts of number of RPA foci per G2 nucleus in 1BR-hTert cells transfected with siCon or siHELLS in the presence or absence of DRB 2 h after 3 Gy IR.  **(E)** Quantification of RPA foci per G2 nucleus 2 h after 3 Gy, in 1BR-hTert cells transfected with Control, HELLS and 53BP1

siRNAs. **(F)** Quantification of BrdU foci per nucleus 1 h after 10 Gy, detected under non-denaturing conditions, in 1BR-hTert cells transfected with Control, HELLS, CtIP and 53BP1

siRNAs. **(G)** Quantification of RPA foci per G2 nucleus 2 h after 3 Gy, in 1BR-hTert cells transfected with Control, HELLS and SMARCAD1 siRNAs. \*\*\*\* indicates a p-value of <0.0001,

\*\*\* indicates p-value of <0.001, \*\* indicates p-value of <0.01, \*\* indicates p-value of <0.1 and ns indicates not significant by unpaired two-tailed t-test analysis. For panels A, C, D, E, F and G, data shown are the mean and sem from three independent experiments.

**Figure S5. HELLS interacts with CtIP and contributes to its accumulation at IR-induced** **breaks**

**(A)** Individual counts of number of CtIP-GFP per nucleus 4 h following 3 Gy IR in U2OS cells transfected with CtIP-GFP expressing plasmid and the indicated siRNA. **(B)** Coomassie stained SDS-PAGE gel showing 500 ng of purified recombinant His-HELLS and CtIP proteins.

**(C)** Individual counts of the number of IR-induced CtIP-GFP foci per nucleus in U2OS cells transfected with CtIP-GFP expressing plasmid, HELLS siRNA and constructs expressing siRNA-resistant wt or ATPase mutant HELLS. **(D)** Western blot analysis of myc-HELLS wt and myc-HELLS K254R proteins in chromatin fraction of U2OS cells transfected with the indicated siRNA and plasmid. **(E)** Quantification of clearance of γ-H2AX foci in G2 nuclei following 3 Gy IR in 1BR-hTert cells transfected with Control, CtIP and HELLS siRNAs. **(F)** Individual counts of number of γ-H2AX foci per G2 nucleus following 3 Gy IR in 1BR-hTert cells transfected with Control, CtIP and HELLS siRNAs. \*\*\*\* indicates a p-value of <0.0001, and ns indicates not significant by unpaired two-tailed t-test analysis. In panels A, C, E and F data were combined from three independent experiments with the mean and sem shown.

**Appendix**

**siRNA oligonucleotide sequences**

The following siRNA oligonucleotides were purchased from Santa Cruz: control siRNA (non-targeting negative control) (sc-37007) HELLS siRNA (LSH) (sc-46665)

The following siRNA oligonucleotide were purchased from Thermo-Fisher Scientific: DNMT3b (4427038, ID: s4221)

The following siRNA oligonucleotides were synthesised by Eurofins: KAP-1, 5’-GCAUGAACCCCUUGUGCUGUUUUGU-3’

CtIP, 5’-GCUAAAACAGGAACGAAUC-3’

DNMT1, 5’- AUUACGUAAAGAAGAAUUA -3’

53BP1, 5'-AGAACGAGGAGACGGUAAUAGUGGG-3'

SMARCAD1, 5'-GAGAUGUAGUUAUAAGGCUUAUGAA-3'

HELLS, 5'-GAUAGAGAGUCGACAGAAA-3'

The following siRNA oligonucleotide was synthesised by Eurogentec: BRCA1, 5’-GGAACCUGUCUCCACAAAG-3’

**Antibodies**

The following antibodies were used for Western blot analysis: Primary antibodies:

1:100 anti-LSH (H4) (for detection of HELLS) (sc-46665, Santa Cruz), 1:1000 α-tubulin (DM1A) (sc-32293, Santa Cruz), 1:500 anti-CtIP (61141, Active Motif), 1:5000 anti-histone H3

(ab1791, Abcam), 1:1000 anti- DNMT1 (D63A6) (XP #5032, Cell Signaling), 1:500 anti-DNMT3b (G-9) (sc-376043, Santa Cruz), 1:1000 anti-phospho KAP-1 (S824) (A300-767A, Bethyl Laboratories), 1:1000 anti-H3K9me3 (ab8898, Abcam).

Secondary antibodies:

1:5000 anti-mouse-HRP IgG (A4416, Sigma-Aldrich), 1:10000 anti-rabbit-HRP IgG (31460, ThermoFisher-Scientific)

The following antibodies were used for Immunofluorescence: Primary antibodies:

1:1000 anti-phospho-Histone H2A.X (Ser139) (clone JBW301) (05-636, Merck), 1:5000 anti-c-Myc (clone 9E10) (MABE282, Merck), 1:100 anti-Rad51 (H-92) (sc-8349, Santa Cruz), 1:100 anti-RPA (RPA34-19) (NA18, Merck), 1:200 anti-phospho KAP-1 (S824) (A300-767A, Bethyl Laboratories), 1:100 anti-BrdU (B44) (347580, BD Biosciences), 1:800 anti-CENPF

(ab5, Abcam), 1:50 anti-LSH (H4), (sc-46665, Santa Cruz)

Secondary antibodies:

1:100 anti-mouse-FITC IgG (F0257, Sigma-Aldrich), 1:200 anti-rabbit-Cy3 IgG (C2306, Sigma-Aldrich), 1:500 anti-rabbit-Alexa488 IgG (A-21206, ThermoFisher-Scientific), 1:500

anti-mouse-Alexa555 IgG (A-21424, ThermoFisher-Scientific), 1:1000 anti-mouse-Alexa647

IgG (4410, Cell Signaling)

**FACS analysis**

Cells were fixed in 70% ethanol and washed in PBS. Cell pellet was resuspended in 100 µl RNAse A (100 µg/ml DNase free, ThermoFisher Scientific) and incubated for 5 min at RT. 400

µl 50 µg/ml propidium iodide was added and samples analysed on the flow cytometry device BD Fortessa X-20. Cell cycle distribution was analysed using Flowing 2.5.1 software. For GFP content analysis, percentage of GFP positive cells were determined by analysis of PI (PECF594-A) and GFP (FITC-A). For analysis of HELLS in G1 and G2 cells, samples were incubated with 1:50 dilution of anti-HELLS antibody in 2% BSA/PBS for 1h on ice. Samples were washed and incubated with 1:100 FITC-conjugated secondary antibody in 2% BSA/PBS

for 1h on ice. Samples were washed and incubated with 1 µg/ml DAPI in 2% BSA/PBS for 30

min on ice. Samples were analysed on Acea Novocyte using 405 nm and 488 nm lasers.

**Recombinant protein expression and purification** The genes encoding wild-type human CtIP (C-terminal 3C-cleavable StrepII tag) and HELLS

(N-terminal His tag) were cloned into pACEBAC1 vector using the BamHI/XbaI and NcoI/XbaI restriction sites, respectively. These plasmids were subsequently transposed into the Tn7attachment site of MultiBacTurbo bacmid DNA for use in the MultiBac system (Geneva Biotech). Bacmids were transfected into Sf9 cells (5 x 105 cells/mL) using Cellfectin II reagent (Invitrogen) in Insect Express media (Lonza) before viral amplification using standard techniques. For large scale expression, 500 mL of Hi5 cells (2 x 106 cells /mL) were infected with 20 mL of Sf9 virus stock for 72 hours at 27°C, harvested by centrifugation, and stored at -20°C.

For CtIP purification, infected Hi5 cells were resuspended in lysis buffer (50 mM Tris- HCl pH

8.0, 500 mM NaCl, 2 mM DTT, 10% glycerol, 1 mM Na3VO4, 20 mM β-glycerophosphate, protease inhibitor cocktail) and sonicated on ice for a total of 3 minutes. Cleared lysate was applied to Streptactin beads (GE Healthcare) and incubated for 1h at 4°C. Beads were washed with 20 mM Tris-HCl pH 8.0, 500 mM NaCl, 5 % glycerol, 1 mM DTT, and elution performed in the same buffer containing 2.5 mM desthiobiotin. CtIP-containing fractions were diluted to 125 mM NaCl and applied to a 5 mL Heparin column (GE Healthcare). CtIP

was eluted with a gradient from 150 mM to 1 M NaCl in buffer containing 20 mM Tris-HCl pH

8.0, 5 % glycerol, 1 mM DTT. CtIP containing samples were subjected to HRV32 protease cleavage and applied to a streptactin column (Qiagen) to remove the StrepII tag. The flow-through containing untagged CtIP was diluted to 125 mM NaCl and applied to a 1 ml MonoQ

column (GE healthcare). CtIP was eluted with a linear gradient of 125 mM to 500 mM NaCl in 20 mM Tris-HCl pH 8.0, 5 % glycerol, 1 mM DTT. Fractions containing CtIP were pooled and applied to Superdex 200 column in 20 mM Tris-HCl pH 8.0, 200 mM NaCl, 5 % glycerol, 1 mM DTT. Purified CtIP was concentrated using a centrifugal filter unit (Millipore), and stored at -80°C.

For purification of His-HELLS 500 mL infected Hi5 cells were resuspended in lysis buffer (50

mM Tris-HCl pH 8, 300 mM NaCl, 0.1 % Triton X-100, 10 % glycerol, 20 mM imidazole, 1

mM DTT, protease inhibitors). Lysate was sonicated and clarified by centrifugation (15,000

rpm for 20 mins at 4°C). Soluble fraction was applied to 1 ml equilibrated Ni-NTA agarose resin and batch-bound for 2 hours at 4 °C. Beads were washed in lysis buffer containing 50

mM imidazole and bound protein eluted in lysis buffer containing 250 mM imidazole.

Fractions containing HELLS were pooled, dialysed into 50 mM Tris-HCL pH 8, 300 mM

NaCl, 0.1% Triton X-100, 1 mM DTT, 10 % glycerol and applied to a S200 SEC column (GE

Healthcare). Fractions containing His-HELLS fractions were concentrated and stored at -

80°C.

previous page
start
