# Supplementary material for: The human HELLS chromatin remodelling protein promotes end resection to facilitate homologous recombination and contributes to DSB repair within heterochromatin
Source: Nucleic Acids Res. 2019 Dec 5;48(4):1872–85. doi: 10.1093/nar/gkz1146 (PMC7038987; doi:10.1093/nar/gkz1146)
Supplement: gkz1146_Supplemental_Files [file gkz1146_supplemental_files.zip › Revised Supplementary Figures gkz1146.zip!/5137_c9cb052bef23499ebcff067d6e4974e3f533162854f3745589f22786b87412ca.html]

Unknown
- 5137 c9cb052bef23499ebcff067d6e4974e3f533162854f3745589f22786b87412ca
None


# 5137 c9cb052bef23499ebcff067d6e4974e3f533162854f3745589f22786b87412ca

Unknown

## No table of contents present

**begin to read**

next page
